# Active role of participants in neuroempowerment training and supportive neurotechnologies: a theoretical-methodological perspective

**DOI:** 10.3389/fpsyg.2025.1660000

**Published:** 2025-08-11

**Authors:** Benedetta Vignati, Davide Crivelli

**Affiliations:** ^1^Faculty of Psychology, Università Cattolica del Sacro Cuore, Milan, Italy; ^2^International research center for Cognitive Applied Neuroscience (IrcCAN), Università Cattolica del Sacro Cuore, Milan, Italy

**Keywords:** neuroempowerment, wearables, participatory protocols, agency, self-determination, self-efficacy

## Abstract

This perspective paper examines the relevance and implications of fostering an active role for participants in neuroempowerment and applied healthcare research, particularly within remote, home-based intervention protocols. Anchored in key psychological constructs—self-efficacy, self-determination, and agency—we argue that the intentional engagement of individuals in their own self-care and enhancement processes is critical to both intervention success and the ecological validity of research outcomes. These constructs provide a framework through which participants are not only involved but become co-agents in shaping their health trajectories. Advances in mobile health technologies and wearable neurotechnologies, such as neurofeedback and biofeedback systems, may further enable participants to access real-time insights into their physiological and neurocognitive states. These tools translate internal processes into actionable feedback, supporting self-regulation, sustained motivation, and embodied learning. Such interactive feedback mechanisms may help bridging unconscious or pre-reflective bodily signals with conscious awareness, thus promoting neurocognitive autonomy. We propose that empowerment-based interventions—especially those incorporating wearable systems—should be intentionally designed to reinforce autonomy, competence, and relatedness, fostering engagement and long-term behavioral change. This participatory model moves beyond traditional therapist-centered approaches, emphasizing personalized, scalable, and self-directed care. Additionally, we highlight the need for research methodologies that recognize participants as co-constructors of their own empowerment paths, encouraging future studies to adopt inclusive, action-oriented frameworks. By integrating psychological theory, neuroscientific models, and digital health innovations, this perspective outlines a multidimensional approach to neuroempowerment, aimed at promoting self-awareness, agency, and meaningful transformation in both clinical and non-clinical populations.

## 1 Active role of participants in research on neuroempowerment and self-enhancement: fact or myth?

A growing segment of the psychological literature has focused on remote empowerment interventions and home-based treatments involving both clinical and non-clinical populations. These approaches, situated at the intersection of prevention and treatment, are characterized by training protocols, often delivered in participants' homes and built around cognitive, psychological, and/or physical exercises (Farah et al., 2004; Crivelli et al., 2022; Crivelli and Vignati, 2025; Smits et al., 2022). In these paradigms, individuals should be craven to assume an active role in their self-care process, often supported by technological devices or online platforms that reflect a telemedicine approach. One important branch of telemedicine—mobile health (m-health)—has, in particular, facilitated the development of tailored pathways that allow participants—whether patients or healthy individuals (or, at least, supposedly healthy individuals, a key critical aspect of applied healthcare research that we acknowledge as often overlooked)—to autonomously follow psychological and psycho-physical treatments at home, typically aided by technologies such as mobile phones (Changizi and Kaveh, 2017; Faiola and Holden, 2017; Vo et al., 2019; Kang and Exworthy, 2022).

In this perspective article, we would like to focus on what we deem as a core aspects of applied healthcare research—i.e., the role and, especially, the “active role” of participants in the empowerment, self-care, and/or self-enhancement process—and its fundamental components, regardless of the specific research population. At first, we discuss the potential benefits of fostering participants perception and actual enaction of an “active role” in empowerment training protocols. We then start a debate on the current and future potential of wearable devices in supporting such aspects of empowerment and prevention interventions. Finally, we reflect on the complexities and opportunities inherent in active involvement in remote training protocols, considering their potential to foster “inner development” and self-transformation. The active role of participants in healthcare applied research is, indeed, shaped by multiple motivational, psychological and contextual factors. We question whether active participant involvement in empowerment and prevention protocols is necessary to ensure both the enhanced wellbeing of participants and the validity of study outcomes.

A central element of active participation concerns the psychological motivations that drive individuals to engage with healthcare and neuroempowerment. While focusing especially on continuance intention of engaging and using mHealth, (Wang et al. 2022) noted that the top five core factors are attitude toward mHealth, users' satisfaction concerning the product performance, presence of an effective strategy for promoting personal health and perceiving control on health status, perceived usefulness of the protocol, and perceived quality of results with respect to health life. In addition, even the methods employed in data collection may substantially influence active participation. Research designs that incorporate revision cycles, rapid prototyping, and/or iterative feedback—e.g., using surveys followed by qualitative interviews—can strengthen participants' sense of agency and relevance, fostering deeper engagement (Zogas et al., 2024). Psychological interventions that explicitly encourage participant involvement further support this notion. For example, addressing issues such as insomnia in community settings has shown that increasing participants' intentions to engage leads to better psychological outcomes (Swierzbiolek et al., 2024). Taken together, these findings underscore the need for multifaceted approaches to participant engagement that consider motivational, contextual and methodological dimensions.

## 2 Active role of participants: core components and fundamental features

A proper “active role” of participants in healthcare protocols is underpinned by several psychological constructs that are engaged and potentially enhanced through training. These variables can shape the psychological and cognitive outcomes observed in such studies. Specifically, we here would like to point out the relevance of three key components shaping the role of a participant involved in healthcare applied research: self-efficacy, self-determination and agency. Below, we examine these concepts, beginning with their foundational definitions in psychological science.

### 2.1 Self-efficacy

Self-efficacy, extensively theorized by Albert Bandura, refers to an individual's belief in their ability to execute behaviors necessary to achieve specific outcomes, a belief that plays a crucial role in how goals are approached, ultimately influencing a person's motivation and performance levels in various contexts (Bandura, 1977, 1989, 2001). Bandura identified four primary sources of self-efficacy beliefs: mastery experiences, vicarious experiences, verbal persuasion and physiological-emotional states. Among these, mastery experiences are the most influential, as successfully performing tasks strengthens belief in one's capabilities and reinforces individuals' belief in their ability to perform similar tasks in the future (Ashford et al., 2010). Conversely, repeated failures can diminish self-efficacy. Such mechanism is so strong and innate that vicarious experiences—like observing others succeed or fail—also shape these beliefs (McCarthy et al., 2015; Walter et al., 2019). Verbal persuasion, including supportive feedback from peers or mentors, and physiological and emotional states can further reinforce self-efficacy, motivating individuals to attempt new challenges (Warner et al., 2014; Yang et al., 2024), with induced negative effects—such as anxiety—undermining self-efficacy and calm and positive states bolstering it.

Classic empirical research has since consistently demonstrated strong associations between self-efficacy and performance across domains, where individuals with high self-efficacy display greater persistence, resilience, and overall achievement (Schunk, 1995). In sports, for instance, self-efficacy predicts athletes' engagement and success (Gernigon and Delloye, 2003; Zagórska and Guszkowska, 2014; O'Neil, 2023). Likewise, in healthcare promotion and rehabilitation contexts, interventions that foster self-efficacy have been shown to improve adherence to treatment and facilitate healthier lifestyle changes (Ashford et al., 2010; Salemonsen et al., 2020; Yang et al., 2024).

### 2.2 Self-determination

Self-Determination Theory (SDT), originally proposed by Deci and Ryan (1985), pivot around the notion of the self as an active, effective agent with the inherent capacity to self-regulate. SDT provides a comprehensive framework for understanding human motivation, personality development, dedication to self-enhancement and wellbeing. At its core lies the concept of autonomy, or the need to feel in control of one's actions and goals. When individuals perceive their actions as self-determined, they exhibit greater intrinsic motivation, engagement, and satisfaction (Ryan and Deci, 2000; Deci and Ryan, 2008). Research in educational and athletic contexts consistently demonstrates that autonomy-supportive environments promote motivation and enhance performance outcomes (Lewthwaite and Wulf, 2017; Mammadov and Schroeder, 2023; Shi et al., 2025).

A second fundamental need in SDT is competence, defined as the individual's perceived ability to effectively interact with their environment and achieve desired outcomes. Meeting this need has been positively linked to motivation and persistence in diverse fields, including sport and education (Haivas et al., 2012; Wang et al., 2024). Also, creating environments where individuals can demonstrate skills and receive feedback is known to strengthen their sense of competence even in everyday life and applied contexts.

The third need—relatedness—involves feeling connected to others and developing a sense of belonging. Satisfying this need has been associated with increased motivation and psychological wellbeing in various contexts (e.g., Kasser and Ryan, 1999; Krause et al., 2019; Šakan et al., 2020). SDT suggests that when autonomy, competence, and relatedness needs are met, individuals experience greater eudaimonic wellbeing—defined as flourishing and living in accordance with one's ideals (Martela and Sheldon, 2019). Consequently, protocols that support these basic psychological needs are more likely to promote participant motivation and adherence.

### 2.3 Agency

The construct of agency is multifaceted, encompassing individual autonomy, collective efficacy and the interplay of interpersonal dynamics. At its core, agency refers to individuals' capacity to make choices, act intentionally, exert control over their lives, and, especially, to both pre-reflectively self-attribute an agentive stance and reflectively recognize themselves as the agents generating the thoughts and behaviors connoting such choices and actions (Crivelli and Balconi, 2010, 2017). It is a central factor in understanding engagement in behavioral health, psychological wellbeing and processes of empowerment.

Recent research by Kimpah et al. (2024) highlights the relationship between individual cognitive empowerment and work performance, demonstrating that intrinsic motivational factors—often framed as psychological empowerment—are associated with enhanced performance. This underscores the role of personal motivation and awareness in fostering agency. A strong perception of agency has also been linked to increased sense of mastery and control, which is crucial for individual motivation and mental health, contributing to life satisfaction and resilience (Hitlin et al., 2015). Notably, higher agency is associated with improved mental health outcomes, underscoring its relevance even in therapeutic contexts (Adler, 2012). Beyond the individual level, agency also supports collective processes (Fernández-Ballesteros et al., 2002), contributing to the effectiveness of community-based initiatives.

## 3 Participatory role and agentive stance in healthcare: the case of home-based neuroempowerment training

Given the premises above, we stress the centrality of an active participatory role and a proper agentive stance as a driver of engagement and therapeutic efficacy in participants even in the field of applied healthcare research.

In neurorehabilitation, patients' agency is operationalized through mechanisms such as engagement, self-management, and decision-making. Engagement is not a unidimensional construct: it encompasses behavioral, cognitive, and emotional dimensions, each of which contributes to how participants interact with therapeutic activities (Matamala-Gomez et al., 2020). When individuals experience themselves as active agents—capable of influencing the course and content of their rehabilitation—they are more likely to remain committed, sustain effort and internalize goals. Medley and Powell (2010) have demonstrated that techniques such as motivational interviewing, which enhance self-awareness and readiness for change, directly impact rehabilitation outcomes by reinforcing personal agency.

Furthermore, as above-noted, SDT provides a robust framework for understanding how intrinsic motivation and perceived autonomy shape sustained therapeutic engagement. Individuals who feel in control of their healthcare journey, rather than directed by external actors, demonstrate stronger adherence to interventions and greater psychological resilience (Ryan and Deci, 2000; Corrigan et al., 2012; Matamala-Gomez et al., 2020; Simpson et al., 2025). Reorienting an individual's locus of control—through strengths-based or collaborative models—has been shown to foster a proactive stance toward recovery, even in the presence of significant neurological or functional impairments.

This perspective is supported by findings from studies in traumatic brain injury (e.g., Möller et al., 2021), anxiety and stress-related disorders (e.g., Danielsson et al., 2020) and sports rehabilitation (e.g., Chan and Hagger, 2012; Jordan et al., 2025), all of which highlight how enhancing active participation and agency in the healthcare process improves both subjective experience and objective outcomes. The concept of agency also extends into technological contexts, including robotic-assisted rehabilitation, where devices are increasingly being designed not merely as therapeutic tools, but as interactive systems that engage the user's volition and cognitive involvement (Turner et al., 2013). Neuroscientific evidence further supports the relevance of agency in therapeutic settings. Studies using neuroimaging and non-invasive brain stimulation have identified specific neural networks—linked to voluntary action, executive function and decision-making—that underlie the sense of agency (Crivelli and Balconi, 2017; Haggard, 2017). These findings suggest that fostering agency in rehabilitation is not only a psychological imperative but also a neurophysiological one, with implications for optimizing the structure and timing of intervention protocols.

Collectively, this body of work further depose in favor of a shift away from traditional therapist-centered paradigms toward participatory, patient-centered models of care, especially in neuroempowerment-oriented applied research. It is indeed crucial that the neuroempowerment process evolves into a shared space of co-construction, where the participant/patient is not just a recipient of treatment but a collaborator in its design, execution and progress.

Within this framework, home-based neuroempowerment training represents a particularly compelling and scalable application. By transferring the site of care to the participant's living environment and equipping them with tools for autonomous self-management, these interventions exemplify the principles of agency-driven engagement. The next section explores how such programs—across self-enhancement, chronic illness management and beyond—enable individuals to assume a more active role with the support of neurotechnologies, with positive implications for health, wellbeing and the sustainability of care models.

### 3.1 Promoting participation and agency via supportive neurotechnologies in neuroempowerment and applied healthcare research

Relevant for present discussion, the intersection of neuroscience, digital health and rehabilitation science has increasingly emphasized the importance of integrating human agency into intervention design not only through psychological constructs, but also through the affordances of technological tools. Among these, wearable devices have emerged as key enablers of participant engagement, fostering an active role throughout the processes of self-monitoring, feedback-based regulation and personalized adaptation of training routines.

In line with the theoretical foundations discussed in the previous sections, the introduction of wearable technologies represents a practical, scalable and user-centered solution to reinforce participation in healthcare processes, foster participants' agency and promote individualized empowerment protocols—both in laboratory settings and in home-based experimental interventions. These devices not only support the remote delivery of cognitive, psychological and physical training but also operationalize participants' active role by translating abstract constructs (e.g., arousal, attentional focus, autonomic balance) into real-time, interpretable data. By allowing individuals to interact with their psychophysiological states in a meaningful way, wearable tools help sustain motivation and cultivate self-awareness, two critical precursors to successful empowerment (Balconi et al., 2017; Balconi and Crivelli, 2019; Crivelli et al., 2022). Research in this area has increasingly demonstrated how technology-mediated training protocols can significantly improve outcomes related to wellbeing, emotion regulation, and cognitive performance [(Bhayee et al., 2016; Colzato et al., 2017; Davis, 2017; Dessy et al., 2018; Stephenson et al., 2017; Balconi and Crivelli, 2019; Balconi et al., 2019; Crivelli et al., 2019b,a; Smith et al., 2020; Kang and Exworthy, 2022; Khosravi et al., 2022; Barac et al., 2024) but, for critical notes, see also (Butcher, 2003; Farah et al., 2004; Nagel, 2014; Martins et al., 2017)]. These technologies play a dual role: on the one hand, they provide assessment and monitoring functions; on the other, they serve as facilitators of empowerment, by offering direct support for behavior change, self-regulation and training adherence.

Two main categories of wearable devices have been widely adopted in this context: neurofeedback and biofeedback systems. These technologies enable individuals to observe, understand and actively regulate their internal states, enabling the possibility to foster mastery, competence, and self-efficacy in both clinical and non-clinical contexts via improved self-awareness and self-regulation. By externalizing physiological and neurocognitive signals, they transform abstract internal processes into actionable data, making them accessible targets for personal development and behavioral adaptation. Neurofeedback systems measure real-time brain activity, typically using non-invasive electroencephalographic (EEG) sensors placed on the scalp. These systems can detect patterns of cortical activation associated with attention, cognitive workload, and relaxation. Through digital platforms, users receive feedback—often visual or auditory—based on their neural activity while performing tasks such as meditation, focused attention, or emotional regulation. This closed-loop interaction supports the development of cognitive self-awareness and allows individuals to train specific mental states, such as sustained attention or calmness, through repeated feedback and adjustment. Biofeedback systems, by contrast, focus on peripheral physiological signals regulated by the autonomic nervous system, including heart rate variability, skin conductance, respiratory patterns, and muscle tension. These parameters are measured through wearable sensors placed on the skin and are used to reflect real-time levels of arousal, stress, or autonomic balance. The feedback allows users to identify maladaptive physiological responses—such as heightened arousal under stress—and to practice strategies aimed at restoring homeostasis, such as slow-paced breathing or progressive muscle relaxation.

Despite targeting different physiological systems, both neurofeedback and biofeedback contribute to a shared goal: enhancing the user's capacity for self-regulation through real-time interaction with their body and brain states. When embedded in structured empowerment protocols—especially those delivered in home-based or hybrid settings—these technologies may foster engagement, autonomy and sustained motivation. Users are not only recipients of interventions but also active participants who learn to recognize, interpret, and modulate their own physiological and cognitive responses.

Importantly, wearable feedback systems reinforce key psychological constructs associated with active role, including self-efficacy, agency and self-determination. By offering immediate and tangible evidence of one's ability to influence internal processes, these tools may help cultivating a sense of control and competence that is essential for long-term behavior change. Moreover, the accessibility and adaptability of wearable technologies make them well suited for personalized, scalable interventions across diverse populations and goals—from stress reduction and cognitive training to emotional self-regulation and performance enhancement.

We therefore propose that supportive neurotechnologies, if properly integrated in applied healthcare and remote neuroempowerment protocols, are not merely instruments of measurement but may become active facilitators of change. Their integration into self-enhancement and neuroempowerment protocols enables a shift from externally driven care to intrinsically motivated engagement, advancing a more participatory and agency-centered model of psychological and psychophysiological intervention.

## 4 Discussion

The promotion of an active role in psychological and neurocognitive research marks a decisive shift toward interventions centered on personal autonomy, intentionality, and self-regulation. As outlined throughout this review, the constructs of self-efficacy, self-determination and agency form a synergistic triad that supports individuals in becoming active agents in their own wellbeing. These constructs are not merely theoretical abstractions but are substantiated by neuroscientific and psychological models that highlight the embodied, experiential nature of self-regulation and empowerment.

In this light, the integration of supportive neurotechnologies such as neurofeedback and biofeedback may become particularly relevant. These tools externalize and render accessible internal states—cognitive, emotional, and physiological—that are otherwise processed implicitly or pre-consciously. Their utility aligns with the model proposed by Balconi et al. (2017), which emphasizes the dynamic flow between unconscious, pre-conscious and conscious processes in shaping emotional and behavioral responses. Specifically, wearable feedback systems facilitate a shift from implicit, automatic patterns of regulation to more explicit and intentional strategies by reinforcing attentional control and interoceptive awareness. This shift is also central to the notion of neurocognitive autonomy, a concept implicitly tied to agency. As Balconi et al. describe, autonomy is not only a motivational construct but a neurobiologically grounded capacity to self-initiate and direct behavior in the absence of external coercion. Neurofeedback and biofeedback foster this form of autonomy by promoting real-time learning based on endogenous feedback—what the authors conceptualize as a form of implicit learning progressively brought under conscious control. Through repeated exposure to self-regulatory tasks and feedback loops, participants develop an internal model of their own physiological and neural functioning, enhancing the degree to which their actions become volitional and goal-directed.

Moreover, the active role and the agentive attitude, in this context, should not be seen as fixed traits but as plastic and malleable perspectives, dependent on the recursive interaction between perception, body feedback and affective salience. Participants' engagement is pivotal for every protocol trying to foster self-enhancement and care of own health. In technology-supported neuroempowerment training, feedback is not only informational—it may become emotionally meaningful. It connects the subject to their bodily and mental states in real time, increasing the motivational weight of the task and facilitating learning through embodied experience. Such mechanisms reflect a broader integrative model of neuroempowerment, in which conscious agency emerges from a continuous interaction between lower-level (e.g., autonomic or emotional) and higher-order (e.g., reflective, metacognitive) processes. We pose that healthcare protocols that embrace this complexity—by supporting autonomy, leveraging implicit learning and engaging multiple levels of the self—hold greater potential for sustainable change. This perspective repositions individuals not only as recipients of intervention but as co-constructors of their own empowerment trajectories. In future research, this perspective could be further supported by participatory methodologies such as co-design and participatory action research, where individuals contribute to defining goals, selecting tasks and refining assessment strategies (Cornish et al., 2023). Such approaches would honor the layered, recursive nature of “active participation” itself.

## Data Availability

The original contributions presented in the study are included in the article/supplementary material, further inquiries can be directed to the corresponding author.
